# Technology-based self-management interventions for women with breast cancer: a systematic review

**DOI:** 10.4069/kjwhn.2023.09.07

**Published:** 2023-09-26

**Authors:** Hae Jeong An, Sook Jung Kang, Goh Eun Choi

**Affiliations:** College of Nursing, Ewha Womans University

**Keywords:** Breast neoplasms, eHealth, mHealth, Self-management, Technology

## Abstract

**Purpose:**

Since technology-based interventions can facilitate convenient access to healthcare for women with breast cancer, it is crucial to understand innovative approaches to maintaining the effectiveness of these interventions. Therefore, we conducted a systematic review of technology-based self-management interventions for women with breast cancer in six countries. We analyzed the characteristics of these interventions and examined their diverse health outcomes.

**Methods:**

Six databases were systematically searched to extract research articles using the keywords “breast cancer,” “technology,” and “self-management.” The search was carried out up until June 12, 2023. From the 1,288 studies retrieved from the database search, 10 eligible papers were identified based on inclusion/exclusion criteria. Two authors independently extracted and compared the data from these articles, resolving any discrepancies through discussion.

**Results:**

Most of the 10 studies utilized web- or mobile-based technology, and one used artificial intelligence-based technology. Among the 12 health-related outcome variables, quality of life and symptom distress were the most frequently mentioned, appearing in six articles. Furthermore, an analysis of the intervention programs revealed a variety of common constructs and the involvement of managers in the self-management intervention.

**Conclusion:**

Incorporating key components such as self-management planning, diary keeping, and communication support in technology-based interventions could significantly improve the self-management process for breast cancer survivors. The practical application of technology has the potential to empower women diagnosed with breast cancer and improve their overall quality of life, by providing timely and sustainable interventions, and by leveraging available resources and tools.

## Introduction

Various factors, including advancements in treatment methods, early detection screenings, and improved surgical techniques, have contributed to a notable increase in breast cancer survival rates. In the United States, the 5-year survival rate for breast cancer patients was 91% in 2018 [1]. Meanwhile, in South Korea, the rate rose from 77.9% between 1993 and 1995 to 93.8% between 2016 and 2020 [2]. With these trends, breast cancer survivors may live for many decades. Therefore, it is crucial to develop strategies aimed at enhancing their quality of life while effectively managing the risks of recurrence and mortality.

Breast cancer treatment can lead to psychological and emotional side effects. These not only negatively affect the patient’s quality of life but also result in increased economic burdens, such as productivity losses. As such, it is crucial to provide ongoing interventions and management to support breast cancer patients during and after their treatment [3]. Encouraging self-care and implementing programs that promote healthy lifestyle habits can enhance health outcomes, boost self-efficacy, and lower the risks of cancer recurrence and mortality [4]. Exercise and diet play a significant role in maintaining a healthy lifestyle, which is essential for the well-being of breast cancer patients. Therefore, interventions designed to improve lifestyle habits, including various exercise routines and dietary changes, have been introduced [5,6]. However, these interventions tend to have only short-term effects, highlighting the need to devise strategies that extend their benefits [7,8].

Since the onset of the coronavirus disease 2019 pandemic, eHealth platforms such as websites and video conferencing have seen rapid expansion [9,10]. Telemedicine has provided scalable and flexible methods for healthcare support, effectively replacing many in-person appointments and ensuring continuity of care [11]. Technology-based interventions involve the use and implementation of technological tools or methodologies in the design, development, and distribution of healthcare solutions to target participants [12]. These interventions also utilize readily accessible devices like smartphones, the internet, or mobile sensors to monitor, reinforce, or provide convenient and cost-effective healthcare services to individuals in need of medical care [12]. Notably, technology-based interventions have demonstrated high patient preference in terms of acceptability and utility, fostering patient-centered care through standardization [13]. They also allow breast cancer survivors to share their experiences and provide immediate feedback to healthcare professionals, facilitating real-time interaction [14]. Therefore, technology-based interventions offer patients a convenient and engaging way to continuously receive health assessments, education, symptom management, self-management enhancement, and psychosocial support [15,16].

Moreover, various technologies, such as online chat platforms, diary writing, video-based education, video games, websites, social media platforms, and mobile applications, have been utilized to promote health and provide psychosocial support for breast cancer survivors [17,18]. Given the intensive and long-term treatment required by breast cancer patients, along with their self-management needs, the provision of timely care is crucial for improving overall self-management in this group [19,20]. Consequently, these technology-based self-management interventions allow breast cancer survivors to access timely and effective treatments. They offer a broad array of resources and tools that can improve their health outcomes and foster their psychosocial well-being [21-23].

The effects of mobile health interventions on physical activity and patient-reported health outcomes, such as quality of life, stress, fatigue, and sleep, in patients with breast cancer have been increasingly examined through systematic reviews [24,25]. However, many of these studies have concentrated on a single type of technology or health outcome, making it challenging to assess the intervention’s acceptability among the target population and the overall trends in which variables self-management programs have been applied and their effectiveness. Consequently, a study that includes various technology-based interventions, such as mobile health, eHealth, and artificial intelligence (AI), and evaluates functionality acceptance, utility, engagement, and long-term management should be undertaken. This would provide the necessary information for developing innovative technology-based interventions and minimizing unnecessary costs [26]. The variety in intervention approaches and outcome assessments in technology-based self-management interventions for breast cancer complicates comparisons, and systematic literature reviews that include interventions using multiple media are scarce. Therefore, this systematic review was conducted with the aim of improving our understanding of technology-based self-management interventions and assessing various health outcomes during and after treatment, thereby providing directions for future research. The findings of this study will contribute to the foundational knowledge of intervention development by understanding the characteristics and outcomes of technology-based self-management interventions for women with breast cancer. The review questions were:

1. What do technology-based self-management programs consist of, and what do they provide to women with breast cancer?

2. What outcomes have been evaluated among women with breast cancer after self-management programs?

3. What is the structure of technology-based self-management programs?

## METHODS

### Study design

This systematic literature review, which focused on technology-based self-management programs for women with breast cancer, adhered to the Preferred Reporting Items for Systematic Reviews and Meta-Analyses (PRISMA) 2020 guidelines [27].

### Eligibility criteria

To clarify the inclusion criteria and devise an effective search strategy, we utilized the PICO (Population/Intervention/Control/Outcome) framework [28]. The population for this study comprised women diagnosed with breast cancer, specifically those undergoing treatment and survivors who had completed treatment. The intervention involved a technology-based approach incorporating elements of self-management. This systematic review did not employ a comparison group. While the outcome was not restricted, it was necessary to measure one or more quantitative outcomes to assess the effectiveness of the self-management interventions. We included studies written in either English or Korean that were published in peer-reviewed academic journals.

In this context, technology-based interventions refer to the application of information communication technologies in facilitating the delivery of education and care for health-related conditions [28]. These interventions can be broadly categorized into two types: internet-based and mobile-based [29]. Therefore, in this study, technology-based interventions encompass all internet- and mobile-based technologies utilized in providing health-related information and care to women diagnosed with breast cancer.

Studies were excluded if they: (1) were review papers, editorials, case studies, or protocols, (2) did not specifically address women’s health in relation to breast cancer, and (3) failed to provide detailed information about the intervention.

Our primary outcome of interest was symptoms directly associated with the disease. Secondary outcomes included aspects of psychological health such as quality of life, depression, and anxiety, among others. Physical health factors, including fatigue, diet, and physical activity, were also of interest. Additionally, we considered other health-related outcomes, such as medication compliance.

### Search strategy

From May 23 to June 12, 2023, two researchers comprehensively retrieved studies in five English databases and two Korean databases. These databases included PubMed, the Cumulative Index to Nursing and Allied Health Literature, PsycINFO, Web of Science, Cochrane Central Register of Controlled Trials, Research Information Sharing Service, and Data Base Periodical Information Academic (Appendix 1). The researchers used the following keywords for each database: (breast cancer) AND (mobile OR m-health OR e-health OR web OR app* OR technology-based OR artificial intelligence OR AI OR chatbot OR telehealth OR digital health) AND (self-management OR self-help OR self-care OR self-guided OR self-administ*) AND (program OR intervention). There were no restrictions on the publication date, and search sets were combined using Boolean operators. Additionally, the researchers conducted backward and forward searches of the identified publications to locate other relevant materials.

### Study selection and data extraction

**Figure 1.**
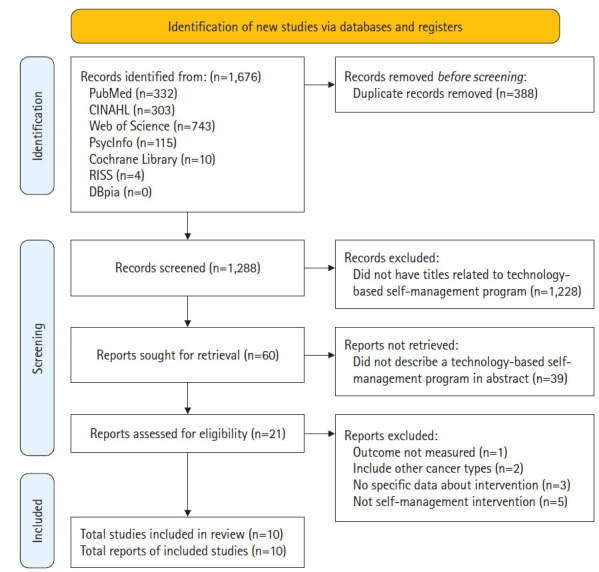
PRISMA 2020 flow diagram of the literature search

Studies were selected in accordance with the PRISMA guidelines. Following the pooling of literature search results, any duplicates were eliminated. Two independent reviewers assessed the titles and abstracts based on the inclusion and exclusion criteria of the studies. Subsequently, the full text of potentially relevant studies was reviewed by two individuals, who then made the selection. Any disagreements between the reviewers were resolved through discussion. The number of studies excluded, along with the reasons for their exclusion, were recorded in a PRISMA flowchart, as depicted in Figure 1.

Using a Microsoft Excel template, two reviewers extracted data from each of the listed studies. The detailed information included the first author, publication year and country, study design, sample/population, sample size, main intervention, control group, study outcome, main findings, and intervention characteristics (technology, program contents, duration, session, intervention manager, intervention manager involvement, and follow-up). The extracted data were subsequently cross-verified to ensure the accuracy of the data extraction process.

### Assessment of risk of bias

Two independent reviewers assessed the quality of the studies included in this review. Any disagreements that arose were resolved through discussion. Specifically, the quality of randomized controlled trials (RCTs) was evaluated using the Cochrane Risk of Bias 2.0 (RoB 2.0) tool. For nonrandomized interventional studies, the Risk of Bias in Nonrandomized Studies-of Interventions (ROBINS-I) tool was employed. The RoB 2.0 tool’s evaluation algorithm was used for each domain to determine whether there was a “low risk,” “some concerns,” or “high risk” of bias. The ROBINS-I tool identified five categories of bias risk across seven domains: “low risk of bias,” “moderate risk of bias,” “high risk of bias,” “very high risk of bias,” and “no information.”

## RESULTS

### Characteristics of selected studies

Out of 1,288 studies identified through an electronic database search, 10 (Appendix 2) were included in this review following a full-text screening, which were named from A1 to A10 [30-39]. Studies with unmeasured outcomes or those that did not focus on self-management interventions were excluded (Figure 1). Three of the studies (A2, A5, and A8) were conducted in the United States, two (A1 and A10) in Europe (specifically the Netherlands and Norway), three (A3, A4, and A7) in East Asia (South Korea), and two (A6 and A9) in the Middle East (Egypt and Iran). Seven of the included studies were RCTs, two were nonrandomized interventional (i.e., quasi-experimental) studies (A6 and A7), and one was a cross-sectional study (A5). The sample sizes ranged from 24 to 355. The control intervention was primarily usual care (80%), with two studies providing an educational booklet (A3 and A4). In the majority of the studies (70%), the follow-up period was identical to the program duration, and the post-test was conducted immediately after the program’s conclusion (A2, A3, A4, A5, A6, A8, and A9). Three studies followed up on program outcomes at 2, 3, and 6 months after the program ended (A1, A7, and A10).

### Risk of bias

**Figure 2.**
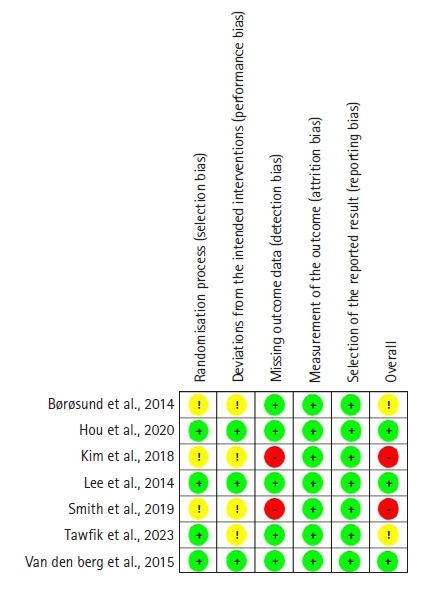
Risk of bias summary of randomized controlled trials using the Cochrane Risk of Bias 2.0 (RoB 2.0) tool.

Figure 2 shows the methodological quality of the RCTs. Using the RoB 2.0 tool, we conducted a Cochrane risk of bias analysis on the RCTs. Three of the seven RCTs did not report allocation concealment (A1, A3, and A8), which raised potential concerns regarding selection bias. Given the inherent characteristics of psychosocial interventions, it is challenging to blind participants, which inevitably resulted in performance bias in four studies (A1, A3, A8, and A9). Two studies failed to detail their approach to handling missing data (A3 and A8), thereby elevating the risk of detection bias. However, no significant attrition bias or reporting bias was observed.

**Figure 3.**
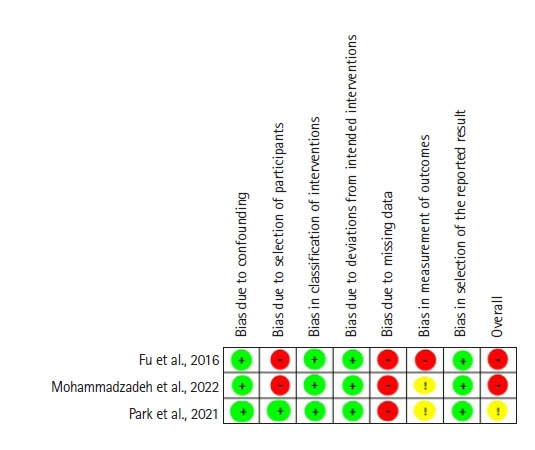
Risk of bias summary of non-randomized controlled trials using the Risk of Bias in Nonrandomized Studies-of Interventions (ROBINS-I) tool.

Figure 3 depicts the methodological quality of non-RCTs. Using the ROBINS-1 tool, we conducted a Cochrane risk of bias analysis on the non-RCTs. All three non-RCTs demonstrated a high risk of detection bias due to the absence of a description of how dropouts and missing data were handled (A5, A6, and A7). Furthermore, the absence of a control group or a well-defined description of the control group presented a high risk for the classification of the intervention (A5 and A6).

### Technology

The interventions could be broadly categorized into two primary types: those that relied on internet platforms and those that depended on mobile platforms. Of the 10 articles included in our final sample (Table 1), five utilized mobile-based interventions (A2, A3, A5, A6, and A7), four employed web-based interventions (A1, A4, A8, and A10), and one used a web-based intervention that incorporated a chatbot (A9). A common theme across these studies was the assertion that technology-based interventions, such as web-based and mobile-based programs, can provide patient-centered care. This allows patients to evaluate their symptoms at any time and from any location. A unique feature of the mobile-based programs was the inclusion of an innovative training system that used avatar simulation videos to help breast cancer survivors develop self-care skills (A5). Additionally, the web-based program that utilized an AI chatbot offered personalized education tailored to women’s needs. This allowed women to engage in individual conversations and receive customized information based on their specific questions (A9).

### Programs

#### Constructs

The results identified several common constructs in the self-management programs (Table 2). The most commonly observed constructs were those related to the provision of information and communication to assist patients in effectively managing their condition (A1 and A2). Constructs associated with assessment, as well as planning with a diary to improve patients’ knowledge, encourage proactive actions, and efficiently monitor their progress, were also prevalent (A1, A3, A4, and A7). Numerous programs focused on specific symptom management and self-care strategies, aiming to equip patients with the necessary skills and knowledge to manage their symptoms and overall health effectively (A5, A6, and A7). Additionally, a handful of programs incorporated cognitive-behavioral therapy and cognitive reframing to offer personalized support, foster active participation, and enhance patients’ comprehension and coping abilities (A8 and A10).

#### Manager involvement

Five of the 10 studies incorporated the involvement of intervention managers during the interventions (A1, A3, A6, A7, and A8). The strategies varied among the studies. In one study, users were given access to a “Contact Us” section within the app, which allowed them to pose questions at any time (A6). Another study conducted weekly interviews with participants via cell phone throughout the duration of the study. This consistent communication enabled healthcare personnel to continuously monitor and support the patients, providing a uniform and personalized intervention experience (A3). A different strategy involved offering a platform for patients to seek assistance from healthcare personnel at their treatment hospital. This platform enabled patients to ask questions, share experiences, and receive advice from oncology nurses. If necessary, the nurses could also direct further inquiries to physicians and social workers, ensuring comprehensive support and expertise (A1). In another study, participants received regular weekly feedback via email, which encouraged them to consistently engage with the website, learn about self-management, and maintain their health diary. This continuous communication and feedback loop was designed to foster active participation and adherence to the intervention among patients (A7). In a separate study, an online group meeting was facilitated by masters who were equipped with a prepared and certified Pillar Guide (A8).

#### Health-related outcomes

Table 3 lists the health-related outcome variables in the 10 self-management programs. For women with breast cancer, these 10 articles discussed 12 health-related outcome variables. With respect to the primary outcome, the program exhibited positive effects in diminishing pain symptoms (A5, A6, and A8) and mitigating distress and side effects (A1, A3, A5, A9, and A10). It also effectively managed menopausal symptoms (A7). Regarding psychological health, a secondary outcome, the program yielded encouraging results in reducing anxiety (A1 and A4), depression (A1, A4, and A8), fear of cancer recurrence (A10), and in improving quality of life (A2, A3, A5, A6, and A7), self-efficacy (A4, A8, and A10), and empowerment (A10). In terms of physical health, the program was beneficial in alleviating fatigue (A4, A6, A8, and A10) and enhancing dietary quality (A4). Additionally, the program showed positive outcomes in relation to medication compliance (A3).

## DISCUSSION

This systematic review examined technology-based self-management programs designed for supportive care in women with breast cancer, focusing on their content and outcomes. Generally, the methods employed in these self-management programs are somewhat limited, and there is a broad range of variation in both content and outcome variables across different studies. Nevertheless, this review can offer guidance on the factors that should be considered when developing and implementing more effective technology-based self-management programs for women with breast cancer.

Web-based technology currently dominates the field of self-management programs for women with breast cancer, with mobile technology, utilizing devices such as smartphones and tablets, coming in second. Web-based intervention programs have long been favored for their ability to provide timely information and support when necessary [40,41]. Concurrently, the use of mobile technology in self-management interventions for individuals with chronic diseases is on the rise, due to its accessibility and portability. This technology enables patients to self-monitor their symptoms at any time and place [36,42].

Five of the 10 studies included in this review focused on mobile-based interventions. Of these, four studies employed applications, with the exception of the study of Fu et al. [34], which incorporated avatar simulation videos. Mobile applications are viewed as highly suitable and effective tools for self-management. They offer the ability to monitor not only specific symptoms, but also physiological indicators and daily activities such as diet and exercise [43]. Consequently, mobile applications can be effectively used not only for tracking post-treatment symptoms in women with breast cancer, but also for promoting health. It is suggested that further studies be conducted to monitor daily activities like diet and exercise among women with breast cancer using mobile applications. This could serve as a method for promoting the health of women with breast cancer.

In the study of Tawfik et al. [38], which is the most recently published study among those included, an AI chatbot was used for a self-management intervention. The study found that ChemoFreebot, an AI technology, significantly impacted women’s self-care behaviors and mitigated chemotherapy-related side effects [38]. AI chatbots are acknowledged as effective self-management tools, as they can minimize the need for face-to-face consultations and offer further evaluation and self-management advice based on the patient’s response [44]. Considering research that suggests cancer patients require more personalized and tailored information [45], AI chatbots could be a valuable tool for enhancing self-management. While there is still some technical work to be done, AI chatbots hold promise as a healthcare tool and signify a substantial technological advancement [44]. Currently, this self-management program primarily employs web and mobile technologies, but it is progressing by integrating newly developed technologies. Therefore, it is recommended to further develop a self-management program using AI to demonstrate its effectiveness. Moreover, the effectiveness of AI-based interventions should be compared with web/mobile-based self-management programs to determine which technologies can most effectively deliver self-management programs.

The outcomes assessed by self-management programs for women with breast cancer can be categorized into physical symptoms and psychological factors associated with the disease. Physical symptoms, such as menopausal symptoms, fatigue, and pain, showed considerable variation across the studies included. Among the psychological factors, quality of life was the most frequently measured. However, the instruments used to gauge quality of life varied significantly across the studies [31,34,35], complicating the comparison of results. Despite this, the implementation of self-management programs has consistently demonstrated an improvement in quality of life. To strengthen the evidence of a program’s effectiveness, future studies could employ the same instrument to evaluate quality of life or examine the sustainability of the effectiveness. Following quality of life, self-efficacy was the second most frequently measured factor. According to the transtheoretical model, self-efficacy is a determinant that can instigate behavioral change and ultimately enhance quality of life [46]. This factor also serves as a crucial psychosocial resource for self-management programs to be effective for participants [37]. Therefore, self-efficacy is not only a factor that can be positively influenced by self-management programs, but it is also a key determinant for participants to maintain self-management and carry out positive changes.

Regarding the constructs of self-management programs, we found that despite variations in specific program structure and content across different studies, several elements were consistently present. These elements encompassed the provision of information via educational materials, symptom self-management, plan creation, and the provision of psychological support through communication. The integration of these constructs into self-management programs is intended to empower patients, enhance their knowledge and skills, improve symptom management, and foster overall well-being. It is noteworthy that the use of a health diary as a self-management tool effectively bolstered self-efficacy in self-management [30,33,36]. This health diary incorporated self-management strategies, goal-setting activities, and a self-report form [36]. Participants were encouraged to evaluate their daily execution of health-enhancing behaviors and record the extent of their implementation as part of their self-management process [30,33]. This, in turn, motivated them to refine their behaviors and adopt healthier lifestyle patterns [33]. The use of a health diary played a significant role in enhancing self-efficacy in self-management. Its purpose aligns with the objectives of self-management programs, which are to motivate patients to self-manage by enhancing their disease understanding, and to enable them to monitor their health changes and respond appropriately. Given that programs incorporating these elements effectively reduce symptoms and improve self-management, it may be beneficial to consider these elements when designing self-management programs.

Another interesting finding of this review is that the programs’ structure incorporated a communication component, which enabled patients to share their experiences. In one study, patients had the opportunity to participate in online forum discussions, allowing them to anonymously exchange messages with other patients or use a blog platform. This feature provided patients with the reassurance that someone was available to address their concerns, thereby offering psychological support [34]. In a similar vein, another study included a community section where patients could share their thoughts and experiences [36], and senior survivors and healthcare professionals could distribute uplifting information [31]. Social support plays a pivotal role in the life of a cancer patient, potentially transforming their lives by bolstering their will to live [47]. Therefore, patient-to-patient communication within the self-management program could be a key factor in enhancing the program’s effectiveness.

In some studies, intervention managers utilized a variety of strategies, such as being continuously available, conducting regular interviews, and providing consistent feedback throughout their involvement in the program. However, many of the studies included did not involve the intervention manager at any stage in the program, instead allowing participants to navigate the program independently. The results indicated a significant positive impact on outcomes immediately following the intervention program [31,35]. Shi et al. [48] conducted a systematic review of mobile-based self-management programs for symptoms related to chemotherapy in breast cancer patients undergoing treatment and found that a self-management program without an intervention manager had a significantly positive effect. This aligns with our findings, suggesting that a well-structured self-management program can be effective for participants without the need for advice or feedback from an intervention manager. However, Harrington [49] argued that the “involvement of the intervention manager” is a crucial factor in enhancing participants’ health-related outcomes in self-management programs. Support from healthcare providers is reported to be particularly necessary for application-based interventions. Therefore, further research is required to determine whether the effectiveness of the self-management program varies depending on the need for an intervention manager.

This study has several limitations. First, the outcomes varied across the studies, and the tools used were different. Therefore, caution should be taken in interpreting the results. Second, this review only included studies using quantitative measures; thus, important insights from qualitative research may have been missed. Third, many studies did not describe attrition rates or refusals, raising questions about selection bias.

This systematic review offers insights into the structure, measured outcomes, and effectiveness of technology-based self-management programs for women with breast cancer. The most commonly utilized technologies in these programs are web- and mobile-based; however, there has been a recent trend towards incorporating new technologies. No consistent trends in study outcomes were observed due to the significant variation across studies. Despite this, we identified several promising findings within individual studies, particularly the significance of self-efficacy and key components of self-management programs. It’s also important to consider certain factors when designing a self-management program for women with breast cancer, such as the crucial role of communication and the potential inclusion of an intervention manager. Future research should continue to evaluate and confirm the effectiveness of technology-based self-management programs for women with breast cancer, with the aim of helping these women overcome their physical and psychological challenges and enhance their quality of life.

## Figures and Tables

**Table 1.** Characteristics of the included studies (N=10)

No.	Study	Year	Country	Study design	Sample/population	Sample size	Main intervention (program name)	Control group	Study outcomes 1: primary 2: secondary	Main findings
A1	Børøsund et al. [30]	2014	Norway	RCT	167 breast cancer patients	I (IPPC): 45	I (IPPC): internet-based patient-provider communication service	Usual care	1)	The WebChoice group reported significantly lower symptom distress, anxiety, and depression compared with the usual care group. The IPPC group reported significant lower depression scores compared with the usual care group, but no differences were observed for symptom distress or anxiety. No significant differences in self-efficacy were found among the study groups.
						I (WebChoice): 64	I (WebChoice): Web-based illness management system, which included IPPC		- Symptom distress	
						C: 58			- Anxiety	
									- Depression	
									2)	
									- Self-efficacy	
A2	Hou et al. [31]	2020	United States	RCT	112 breast cancer patients	I: 53	Breast cancer self-management support (BCSMS)	Usual care	QoL	The mean total QoL summary scores were significantly higher among the experimental group versus the control group, respectively, at 3 months.
						C: 59				
A3	Kim et al. [32]	2018	South Korea	RCT	72 breast cancer patients with clinical stage Ⅳ	I: 36	A mobile game-based intervention (ILOVEBREAST)	Educational booklet	1)	The use of the mobile game was associated with lower rates of chemotherapy-related side effects, such as nausea, fatigue, numbness of hand or foot, and hair loss, than the control group. The game group exhibited better QoL during chemotherapy. However, there were no significant differences in terms of depression and anxiety scales.
						C: 40			- Time spent on education	
									- Compliance with medication	
									- Physical side effects	
									- Anxiety	
									- Depression	
									- QoL	
A4	Lee et al. [33]	2014	South Korea	RCT	59 breast cancer patients who had completed primary cancer treatment within 12 months	I: 29	Web-based self-management exercise and diet intervention program (WSEDI)	Educational booklet	1)	Participants who engaged in at least moderate-intensity aerobic activity for at least 150 minutes per week, consumed five servings of F&V each day, and saw general gains in nutritional quality, physical functioning and appetite loss (HRQOL), fatigue, motivational readiness, and self-efficacy
						C: 28			- Exercise and intake of F&V	
									- Dietary quality	
									2)	
									- HRQOL	
									- Anxiety and depression	
									- Fatigue	
									- Stage of change	
									- Perceived self-efficacy	
A5	Fu et al. [34]	2016	United States	Cross-sectional design	355 breast cancer survivors	NA	The-Optipal-Lymph-Flow-health IT system (TOLF)	NA	1)	A significant difference in symptom distress or impact on between breast cancer survivors with lymphedema and those without lymphedema. Themes from the qualitative data included empowerment, high-quality information, loving avatar simulation videos, easy accessibility, and user-friendliness.
									- Symptoms of pain, soreness, aching, tenderness	
									- Number of lymphedema symptoms	
									2) Symptom distress/QoL related to pain and symptoms	
A6	Mohammadzadeh et al. [35]	2022	Iran	Quasi-experimental	24 breast cancer patients	I: 24	Self-management mobile application (NI)	NA	1)	The use of the application showed the most significant changes in QoL, including social avoidance, negative feelings, sexual function, sexual interest, and pain.
						C: None			- QoL (negative feelings, positive feelings, cognitive problems, pain, sexual interest, energy/fatigue, social avoidance, financial problems, benefits, distress-family, appearance, distress-recurrence)	
A7	Park et al. [36]	2021	South Korea	Quasi-experimental	60 breast cancer patients who developed amenorrhea while receiving chemotherapy	I: 27	Self-management- program with an integrative cognitive-behavioral intervention	Usual care	1) Menopausal symptoms	In the intervention group, menopausal symptoms were significantly improved compared to the control group at the follow-up test.
						C: 24			2)	In the follow-up test, the intervention group’s self-efficacy and QoL significantly improved, whereas that of the control group decreased.
									- Self-efficacy	
									- QoL	
									(physical well-being, emotional well-being, functional well-being)	
A8	Smith et al. [37]	2019	United States	RCT	86 adult breast cancer survivors with chronic pain	I: 34	*Reimagine*	Usual care	1)	Reimagine has an effect on depression and fatigue symptoms for breast cancer survivors. Online programs can be a feasible and effective alternative to in-person support.
						C: 52			- Depression	
									- Fatigue	
									2)	
									- Pain severity	
									- Pain interference	
									- Self-efficacy	
									- (User satisfaction)	
A9	Tawfik et al. [38]	2023	Egypt	RCT	150 breast cancer patients	I (ChemoFreeBot): 50	I (ChemoFreeBot): Self-care intervention by interacting with a chatbot	Usual care	1)	Significant differences were found between the three groups in terms of the physical symptom frequency, severity, and distress; the psychological symptoms frequency, severity, distress, and the effectiveness of self-care behaviors.
						I (Education): 50	I (Education): Face-to-face education on self-care techniques to manage chemotherapy side effects		- The frequency, severity, and distress of physical and psychological chemotherapy-related side effects	
						C: 50			2)	
									- Usability of the chatbot	
A10	van den Berg et al. [39]	2015	Netherlands	RCT	135 breast cancer patients who had completed curative primary treatment 2 to 4 months	I: 63	Web-based self-management intervention (BREATH)	Usual care	1)	Intervention group reported significantly less distress than comparison group with a small-to-medium effect size, but empowerment was not affected. There were no between-group differences in primary outcomes during follow-up.
						C: 72			- Distress	
									- Empowerment	
									2)	
									- Negative adjustment (fatigue, helplessness, fear of cancer recurrence)	
									- Positive adjustment (self-efficacy, remoralization, personal control, acceptance)	

C, control group; F&V, fruits and vegetables; HRQOL, health-related quality of life; I, intervention group; NA, not applicable; NI, no information; QoL, quality of life; RCT, randomized controlled trial.

**Table 2.** Description of the interventions (N=10)

No.	Study	Year	Technology	Program construct/contents	Duration	Session	Intervention manager	Involvement of intervention manager
A1	Børøsund et al. [30]	2014	Web-based	1) Symptom assessment	12 months	Access any time as needed	Nurse	Online communication with patients and advice
				2) Advice			Physician	
				3) Information			Social worker	
				4) Communication				
				5) Electronic diary				
A2	Hou et al. [31]	2020	Mobile-based	1) Evidence or knowledge about breast cancer	12 weeks	Access any time as needed	Nurse	None
				2) Exercise and rehabilitation after surgery				
				3) Diet and nutrition for breast cancer patients				
				4) Emotional support to prevent anxiety and depression				
				5) A personal health record for tracking treatment and side effects				
				6) Social resource information				
				7) Experience sharing				
				8) Expert consulting.				
A3	Kim et al. [32]	2018	Mobile-based	1) Education for preventing side effects of anticancer drugs	3 weeks	>30 minutes a day, 3 times per week	Nurse	Interview every week via cell phone
				2) Support for the prevention of side effects of anticancer drugs				
				3) Encouragement of mood and activity				
A4	Lee et al. [33]	2014	Web-based	1) Assessment	12 weeks	Access any time as needed	Nurse	None
				2) Education (tailored information provision)				
				3) Action planning (goal setting, scheduling, keeping a diary)				
				4) Automatic feedback				
A5	Fu et al. [34]	2016	Mobile-based (avatar simulation videos)	1) Building self-care skills based on research-based, easily-integrated-into-daily routine self-care strategies to lessen lymphedema symptom burden	12 weeks	Access any time as needed	NI	None
				2) Symptom evaluation				
				3) Daily lymphatic exercises				
				4) Strategies for an optimal body mass index				
				5) Situational self-care strategies				
A6	Mohammadzadeh et al. [35]	2022	Mobile-based	1) Information acquisition	9 weeks	Access any time as needed	NI	Question and answer via application
				2) Lifestyle management				
				3) Psychological management				
				4) Symptom management				
				5) Change compatibility				
A7	Park et al. [36]	2021	Mobile-based	1) Education and information module	12 weeks	Access any time as needed	Healthcare providers (physicians, breast cancer center coordinators)	Regular weekly feedback by email
				- Showing self-management techniques for menopause-related symptoms and health issues experienced by breast cancer patients with CIA				
				2) Communication module for coaching and providing psychosocial support				
				- Included a self-help group and a community consisting of consultations with healthcare providers				
				3) Health diary for self- management				
A8	Smith et al. [37]	2019	Web-based (online+self-paced videos+live classes)	1) Required activities	18 weeks	Access any time as needed	Masters (prepared and certified Pillar Guide)	Online group meeting
				- Attending one online introductory group meeting,				
				- Viewing videos				
				- Completing cognitive reframing and mind-body exercises				
				2) Curriculum teaches two major skill sets				
				- Solution-focused thinking about stressors				
				- Cognitive reframing				
				3) Mind-body exercises				
				- Such as guided imagery and meditation				
A9	Tawfik et al. [38]	2023	AI-based (chatbot)	1) Dialogue with a chatbot	6 weeks	Access any time as needed	Nurse	None
				- Select from a list of commonly experienced chemotherapy-related side effects and the chatbot then provides a detailed answer				
A10	van den Berg et al. [39]	2015	Web-based	1) Cognitive-behavioral therapy and including information	16 weeks	Access any time as needed	NI	None
				2) Assignment				
				3) Assessment				
				4) Video				

AI, artificial intelligence; CIA, chemotherapy-induced amenorrhea; NI, no information.

**Table 3.** Comparison of the health-related outcomes of the included studies (N = 10)

Health-related outcomes	Categories	A1	A2	A3	A4	A5	A6	A7	A8	A9	A10	Total
Primary outcomes	Symptoms of pain					✔	✔		✔			3
	Symptom distress/side effect	✔		✔		✔				✔	✔	5
	Menopausal symptoms							✔				1
Secondary outcomes	Anxiety	✔		✔	✔							3
	Depression	✔		✔	✔				✔			4
	Fatigue				✔		✔		✔		✔	4
	Fear of cancer recurrence										✔	1
	Compliance to medication			✔								1
	Dietary quality				✔							1
	Self-efficacy	✔			✔			✔	✔		✔	5
	Quality of life		✔	✔	✔	✔	✔	✔				6

## Appendix 1.

Search Strategy

**Table:**

Database		Formula	Number of literatures
PubMed	S1	Advanced / All field / breast cancer	418,433
		Filters: Full text, Journal Article, English	
	S2	Advanced / All field / (mobile) OR (m-health) OR (e-health) OR (web) OR (app*) OR (technology-based) OR (artificial intelligence) OR (AI) OR (chatbot) OR (telehealth) OR (mobile platform) OR (digital health*)	1,575,991
		Filters: Full text, Journal Article, English	
	S3	Advanced / All field / (self-management) OR (self-help) OR (self-care) OR (self-guided) OR (self-administ*)	267,655
		Filters: Full text, Journal Article, English,	
	S4	Advanced / All field / (program) OR (intervention)	
		Filters: Full text, Journal Article, English	
	S5	S1 AND S2 AND S3 AND S4	501
CINAHL	S1	Advanced search, no field selected	32,088
		breast cancer	
		Limiters: Full text	
		Expanders: Apply equivalent subjects	
		Narrowed by Language: English	
		Search modes: Boolean/Phrase	
	S2	Advanced search, no field selected	76,117
		“mobile” OR “m-health” OR “e-health” OR “web” OR app OR “technology-based” OR “artificial intelligence” OR “AI” OR “chatbot” OR “telehealth” OR “mobile platform” OR digital health	
		Limiters: Full text	
		Expanders: Apply equivalent subjects	
		Narrowed by Language: English	
		Search modes: Boolean/Phrase	
	S3	Advanced search, no field selected	20,045
		“self-management” OR “self-help” OR “self-care” OR “self-guided” OR self-administ	
		Limiters: Published Date: 2020.1-2021.3, Full text	
		Expanders: Apply equivalent subjects	
		Narrowed by Language: English	
		Search modes: Boolean/Phrase	
	S4	Advanced search, no field selected	176,635
		“program” OR “intervention”	
		Limiters: Published Date: 2020.1-2021.3, Full text	
		Expanders: Apply equivalent subjects	
		Narrowed by Language: English	
		Search modes: Boolean/Phrase	
	S5	S1 AND S2 AND S3 AND S4	303
Cochrane Library	S1	Advanced search	628
		breast cancer	
		Document type: journal article, Language: English	
	S2	Advanced search	8,738
		(mobile OR (m-health) OR (e-health) OR (web) OR (app*) OR (technology-based) OR (artificial intelligence) OR (AI) OR (chatbot) OR (telehealth) OR (mobile platform) OR digital health*)	
		Document type: journal article, Language: English	
	S3	Advanced search	1,165
		((self-management) OR (self-help) OR (self-care) OR (self-guided) OR self-administ)	
		Document type: journal article, Language: English	
	S4	Advanced search	9,088
		((program) OR (intervention))	
		Document type: journal article, Language: English	
	S5	S1 AND S2 AND S3 AND S4	10
Web of science	S1	Advanced search	653,564
		breast cancer	
		Restrict results by languages and document types: English, journal article	
		Publication Years: 2020-2021	
	S2	Advanced search	1,194,787
		((mobile) OR (m-health) OR (e-health) OR (web) OR app OR (technology-based) OR (artificial intelligence) OR (AI) OR (chatbot) OR (telehealth) OR (mobile platform) OR digital health)	
		Document type: Article, Language: English	
	S3	Advanced search	54,301
		((self-management) OR (self-help) OR (self-care) OR (self-guided) OR self-administ)	
		Document type: Article, Language: English	
	S4	Advanced search	6,207,431
		((program) OR (intervention))	
		Publication date: 2020-2021	
		Document type: Article, Language: English	
	S5	S1 AND S2 AND S3 AND S4	743
PsycINFO	S1	Advanced search / Anywhere	6,592
		breast cancer	
		Record type: Scholarly Journals	
		Language: English	
	S2	Advanced search / Anywhere	130,976
		(“mobile” OR “m-health” OR “e-health” OR “web” OR app OR “technology-based” OR “artificial intelligence” OR “AI” OR “chatbot” OR “telehealth” OR “mobile platform” OR digital health)	
		Record type: Scholarly Journals	
		Language: English	
	S3	Advanced search / Anywhere	36,583
		(“self-management” OR “self-help” OR “self-care” OR “self-guided” OR self-administ)	
		Record type: Scholarly Journals	
		Language: English	
	S4	Advanced search / Anywhere	519,343
		(“program” OR “intervention”)	
		Record type: Scholarly Journals	
		Language: English	
	S5	S1 AND S2 AND S3 AND S4	115
RISS	S1	Advanced search	3,817
		유방암	
		구분: 국내학술논문	
		원문유무: 원문있음	
		Language: Korean	
	S2	Advanced search	83,343
		모바일 헬스 | 애플리케이션 | 서비스 디자인 | 웹기반 | 디지털 기술 | 기술기반 | 인공지능| 모바일플랫폼	
		구분: 국내학술논문	
		원문유무: 원문있음	
		Language: Korean	
	S3	Advanced search	18,873
		증상관리 | 자기관리 | 맞춤형	
		구분: 국내학술논문	
		원문유무: 원문있음	
		Language: Korean	
	S4	Advanced search	218,599
		프로그램 | 중재	
		구분: 국내학술논문	
		원문유무: 원문있음	
		Language: Korean	
	S5	S1 AND S2 AND S3 AND S4	4
DBpia	S1	유방암 | 모바일 헬스 | 애플리케이션 | 서비스 디자인 | 웹기반 | 디지털 기술 | 기술기반 | 인공지능| 모바일플랫폼 AND 증상관리 | 자기관리 | 맞춤형 AND 프로그램 | 중재	0
		Journal Article	

## Appendix 2. Studies included in this review

A1. Børøsund E, Cvancarova M, Moore SM, Ekstedt M, Ruland CM. Comparing effects in regular practice of e-communication and Web-based self-management support among breast cancer patients: preliminary results from a randomized controlled trial. J Med Internet Res. 2014;16(12):e295. https://doi.org/10.2196/jmir.3348

A2. Hou IC, Lin HY, Shen SH, Chang KJ, Tai HC, Tsai AJ, et al. Quality of life of women after a first diagnosis of breast cancer using a self-management support mHealth app in Taiwan: randomized controlled trial. JMIR Mhealth Uhealth. 2020;8(3):e17084. https://doi.org/10.2196/17084

A3. Kim HJ, Kim SM, Shin H, Jang JS, Kim YI, Han DH. A mobile game for patients with breast cancer for chemotherapy self-management and quality-of-life improvement: randomized controlled trial. J Med Internet Res. 2018;20(10):e273. https://doi.org/10.2196/jmir.9559

A4. Lee MK, Yun YH, Park HA, Lee ES, Jung KH, Noh DY. A Web-based self-management exercise and diet intervention for breast cancer survivors: pilot randomized controlled trial. Int J Nurs Stud. 2014;51(12):1557-1567. https://doi.org/10.1016/j.ijnurstu.2014.04.012

A5. Fu MR, Axelrod D, Guth AA, Rampertaap K, El-Shammaa N, Hiotis K, et al. mHealth self-care interventions: managing symptoms following breast cancer treatment. Mhealth. 2016;2:28. https://doi.org/10.21037/mhealth.2016.07.03

A6. Mohammadzadeh Z, Eghtedar S, Ayatollahi H, Jebraeily M. Effectiveness of a self-management mobile app on the quality of life of women with breast cancer: a study in a developing country. BMC Womens Health. 2022;22(1):446. https://doi.org/10.1186/s12905-022-02020-5

A7. Park JH, Jung YS, Kim JY, Bae SH. Mobile web-based self-management program for breast cancer patients with chemotherapy-induced amenorrhoea: a quasi-experimental study. Nurs Open. 2022;9(1):655-665. https://doi.org/10.1002/nop2.1113

A8. Smith SK, MacDermott K, Amarasekara S, Pan W, Mayer D, Hockenberry M. Reimagine: a randomized controlled trial of an online, symptom self-management curriculum among breast cancer survivors. Support Care Cancer. 2019;27(5):1775-1781. https://doi.org/10.1007/s00520-018-4431-7

A9. Tawfik E, Ghallab E, Moustafa A. A nurse versus a chatbot ‒ the effect of an empowerment program on chemotherapy-related side effects and the self-care behaviors of women living with breast Cancer: a randomized controlled trial. BMC Nurs. 2023;22(1):102. https://doi.org/10.1186/s12912-023-01243-7

A10. van den Berg SW, Gielissen MF, Custers JA, van der Graaf WT, Ottevanger PB, Prins JB. BREATH: web-based self-management for psychological adjustment after primary breast cancer--results of a multicenter randomized controlled trial. J Clin Oncol. 2015;33(25):2763-2771. https://doi.org/10.1200/JCO.2013.54.9386

## References

[1] American Cancer Society (2022). Cancer treatment & survivorship facts & figures 2022-2024 [Internet]. https://www.cancer.org/research/cancer-facts-statistics/all-cancer-facts-figures/2023-cancer-facts-figures.html.

[2] National Cancer Center (2022). Annual report of cancer statistics in Korea in 2020 [Internet]. https://ncc.re.kr/cancerStatsView.ncc?bbsnum=638&searchKey=total&searchValue=&pageNum=1.

[3] Lozano-Lozano M, Martín-Martín L, Galiano-Castillo N, Álvarez-Salvago F, Cantarero-Villanueva I, Fernández-Lao C (2016). Integral strategy to supportive care in breast cancer survivors through occupational therapy and a m-health system: design of a randomized clinical trial. BMC Med Inform Decis Mak.

[4] Triberti S, Savioni L, Sebri V, Pravettoni G (2019). eHealth for improving quality of life in breast cancer patients: a systematic review. Cancer Treat Rev.

[5] Singleton AC, Raeside R, Hyun KK, Partridge SR, Di Tanna GL, Hafiz N (2022). Electronic health interventions for patients with breast cancer: systematic review and meta-analyses. J Clin Oncol.

[6] James EL, Stacey FG, Chapman K, Boyes AW, Burrows T, Girgis A (2015). Impact of a nutrition and physical activity intervention (ENRICH: Exercise and Nutrition Routine Improving Cancer Health) on health behaviors of cancer survivors and carers: a pragmatic randomized controlled trial. BMC Cancer.

[7] Chung BY, Oh EH (2017). The effect of diet intervention in breast cancer: a meta-analysis. Asian Oncol Nurs.

[8] Kanera IM, Bolman CA, Willems RA, Mesters I, Lechner L (2016). Lifestyle-related effects of the web-based Kanker Nazorg Wijzer (Cancer Aftercare Guide) intervention for cancer survivors: a randomized controlled trial. J Cancer Surviv.

[9] Rittberg R, Mann A, Desautels D, Earle CC, Navaratnam S, Pitz M (2020). Canadian Cancer Centre response to COVID-19 pandemic: a national and provincial response. Curr Oncol.

[10] Baggott C, Jibb L, Parker R, Stinson J, Linder L, Hinds P, Linder L (2020). Pediatric oncology nursing: pediatric oncology.

[11] Gorini A, Mazzocco K, Triberti S, Sebri V, Savioni L, Pravettoni G (2018). A P5 approach to m-Health: design suggestions for advanced mobile health technology. Front Psychol.

[12] Su Z, Li X, McDonnell D, Fernandez AA, Flores BE, Wang J (2021). Technology-Based interventions for Cancer caregivers: concept analysis. JMIR cancer.

[13] Johnson BA, Lindgren BR, Blaes AH, Parsons HM, LaRocca CJ, Farah R (2021). The new normal? Patient satisfaction and usability of telemedicine in breast cancer care. Ann Surg Oncol.

[14] Eyles H, Jull A, Dobson R, Firestone R, Whittaker R, Te Morenga L (2016). Co-design of mHealth delivered interventions: a systematic review to assess key methods and processes. Curr Nutr Rep.

[15] Helm EE, Kempski KA, Galantino ML (2020). Effect of disrupted rehabilitation services on distress and quality of life in breast cancer survivors during the COVID-19 pandemic. Rehab Oncol.

[16] Fuemmeler BF, Holzwarth E, Sheng Y, Do EK, Miller CA, Blatt J (2020). Mila Blooms: a mobile phone application and behavioral intervention for promoting physical activity and a healthy diet among adolescent survivors of childhood cancer. Games Health J.

[17] Kopp LM, Gastelum Z, Guerrero CH, Howe CL, Hingorani P, Hingle M (2017). Lifestyle behavior interventions delivered using technology in childhood, adolescent, and young adult cancer survivors: a systematic review. Pediatr Blood Cancer.

[18] Sansom-Daly UM, Wakefield CE, Ellis SJ, McGill BC, Donoghoe MW, Butow P (2021). Online, group-based psychological support for adolescent and young adult cancer survivors: results from the recapture life randomized trial. Cancers (Basel).

[19] Glynn LG, Murphy AW, Smith SM, Schroeder K, Fahey T (2010). Interventions used to improve control of blood pressure in patients with hypertension. Cochrane Database Syst Rev.

[20] Ferdinand KC, Yadav K, Nasser SA, Clayton-Jeter HD, Lewin J, Cryer DR (2017). Disparities in hypertension and cardiovascular disease in blacks: The critical role of medication adherence. J Clin Hypertens (Greenwich).

[21] Zhang A, Zebrack B, Acquati C, Roth M, Levin NJ, Wang K (2022). Technology-assisted psychosocial interventions for childhood, adolescent, and young adult cancer survivors: a systematic review and meta-analysis. J Adolesc Young Adult Oncol.

[22] Walsh CA, Rosenberg AR, Lau N, Syrjala KL (2020). Key considerations for advancing the development and testing of mHealth interventions in adolescent and young adult oncology. Psychooncology.

[23] Wiener L, Canter K, Long K, Psihogios AM, Thompson AL (2020). Pediatric psychosocial standards of care in action: research that bridges the gap from need to implementation. Psychooncology.

[24] Dorri S, Asadi F, Olfatbakhsh A, Kazemi A (2020). A systematic review of electronic health (eHealth) interventions to improve physical activity in patients with breast cancer. Breast Cancer.

[25] Singleton A, Raeside R, Partridge SR, Hayes M, Maka K, Hyun KK (2021). Co-designing a lifestyle-focused text message intervention for women after breast cancer treatment: mixed methods study. J Med Internet Res.

[26] Li J, Liu Y, Jiang J, Peng X, Hu X (2021). Effect of telehealth interventions on quality of life in cancer survivors: a systematic review and meta-analysis of randomized controlled trials. Int J Nurs Stud.

[27] Page MJ, McKenzie JE, Bossuyt PM, Boutron I, Hoffmann TC, Mulrow CD (2021). The PRISMA 2020 statement: an updated guideline for reporting systematic reviews. Int J Surg.

[28] Melnyk BM, Fineout-Overholt E (2022). Evidence-based practice in nursing & healthcare: a guide to best practice.

[29] Zhu H, Xiao L, Tu A (2022). Effectiveness of technology-based interventions for improving sleep among children: a systematic review and meta-analysis. Sleep Med.

[30] Børøsund E, Cvancarova M, Moore SM, Ekstedt M, Ruland CM (2014). Comparing effects in regular practice of e-communication and Web-based self-management support among breast cancer patients: preliminary results from a randomized controlled trial. J Med Internet Res.

[31] Hou IC, Lin HY, Shen SH, Chang KJ, Tai HC, Tsai AJ (2020). Quality of life of women after a first diagnosis of breast cancer using a self-management support mHealth app in Taiwan: randomized controlled trial. JMIR Mhealth Uhealth.

[32] Kim HJ, Kim SM, Shin H, Jang JS, Kim YI, Han DH (2018). A mobile game for patients with breast cancer for chemotherapy self-management and quality-of-life improvement: randomized controlled trial. J Med Internet Res.

[33] Lee MK, Yun YH, Park HA, Lee ES, Jung KH, Noh DY (2014). A Web-based self-management exercise and diet intervention for breast cancer survivors: pilot randomized controlled trial. Int J Nurs Stud.

[34] Fu MR, Axelrod D, Guth AA, Rampertaap K, El-Shammaa N, Hiotis K, Scagliola J, Yu G, Wang Y (2016). mHealth self-care interventions: managing symptoms following breast cancer treatment. Mhealth.

[35] Mohammadzadeh Z, Eghtedar S, Ayatollahi H, Jebraeily M (2022). Effectiveness of a self-management mobile app on the quality of life of women with breast cancer: a study in a developing country. BMC Womens Health.

[36] Park JH, Jung YS, Kim JY, Bae SH (2022). Mobile web-based self-management program for breast cancer patients with chemotherapy-induced amenorrhoea: a quasi-experimental study. Nurs Open.

[37] Smith SK, MacDermott K, Amarasekara S, Pan W, Mayer D, Hockenberry M (2019). Reimagine: a randomized controlled trial of an online, symptom self-management curriculum among breast cancer survivors. Support Care Cancer.

[38] Tawfik E, Ghallab E, Moustafa A (2023). A nurse versus a chatbot ‒ the effect of an empowerment program on chemotherapy-related side effects and the self-care behaviors of women living with breast cancer: a randomized controlled trial. BMC Nurs.

[39] van den Berg SW, Gielissen MF, Custers JA, van der Graaf WT, Ottevanger PB, Prins JB (2015). BREATH: web-based self-management for psychological adjustment after primary breast cancer--results of a multicenter randomized controlled trial. J Clin Oncol.

[40] Karakus Z, Ozer Z, Bozcuk H (2022). The effect of web-based education on symptom management and quality of life of patients with lung cancer. Int J Caring Sci.

[41] Ruland CM, Andersen T, Jeneson A, Moore S, Grimsbø GH, Børøsund E (2013). Effects of an internet support system to assist cancer patients in reducing symptom distress: a randomized controlled trial. Cancer Nurs.

[42] Abrahams HJ, Gielissen MF, Donders RR, Goedendorp MM, van der Wouw AJ, Verhagen CA (2017). The efficacy of Internet-based cognitive behavioral therapy for severely fatigued survivors of breast cancer compared with care as usual: a randomized controlled trial. Cancer.

[43] Sharma S, Kumari B, Ali A, Yadav RK, Sharma AK, Sharma KK (2022). Mobile technology: a tool for healthcare and a boon in pandemic. J Family Med Prim Care.

[44] Swanepoel DW, Manchaiah V, Wasmann JW (2023). The rise of AI chatbots in hearing health care. Hear J.

[45] Chua GP, Tan HK (2020). A qualitative approach in determining the patient-centered information and supportive care needs of cancer patients in Singapore. BMJ Open.

[46] Prochaska JO, DiClemente CC (1983). Stages and processes of self-change of smoking: toward an integrative model of change. J Consult Clin Psychol.

[47] Hong SJ, Shin NM (2021). Fear of cancer recurrence in Korean women after breast cancer treatment: a mixed methods study. Eur J Oncol Nurs.

[48] Shi N, Wong AK, Wong FK, Sha L (2023). Mobile health application-based interventions to improve self-management of chemotherapy-related symptoms among people with breast cancer who are undergoing chemotherapy: a systematic review. Oncologist.

[49] Harrington L (2018). From apps to mHealth: informing, interacting, and changing behavior. AACN Adv Crit Care.

